# Sentinel Surveillance of Influenza-Like Illness in Two Hospitals in Maracay, Venezuela: 2006–2010

**DOI:** 10.1371/journal.pone.0044511

**Published:** 2012-09-11

**Authors:** Guillermo Comach, Nimfa Teneza-Mora, Tadeusz J. Kochel, Carlos Espino, Gloria Sierra, Daria E. Camacho, V. Alberto Laguna-Torres, Josefina Garcia, Gloria Chauca, Maria E. Gamero, Merly Sovero, Slave Bordones, Iris Villalobos, Angel Melchor, Eric S. Halsey

**Affiliations:** 1 Laboratorio Regional de Diagnostico e Investigacion del Dengue y otras Enfermedades Virales (LARDIDEV), Instituto de Investigaciones Biomedicas de la Universidad de Carabobo (BIOMED-UC), Maracay, Venezuela; 2 Naval Medical Research Center, Silver Spring, Maryland, United States of America; 3 U.S. Naval Medical Research Unit Six (NAMRU-6), Lima, Peru; 4 Hospital José María Carabaño Tosta, Instituto Venezolano de los Seguros Sociales, Maracay, Venezuela; 5 Hospital Central de Maracay, Corporación de Salud de Aragua (CORPOSALUD ARAGUA), Maracay, Venezuela; 6 Dirección de Epidemiología, Corporación de Salud de Aragua (CORPOSALUD ARAGUA), Maracay, Venezuela; National Institutes of Health, United States of America

## Abstract

**Background:**

Limited information exists on the epidemiology of acute febrile respiratory illnesses in tropical South American countries such as Venezuela. The objective of the present study was to examine the epidemiology of influenza-like illness (ILI) in two hospitals in Maracay, Venezuela.

**Methodology/Principal Findings:**

We performed a prospective surveillance study of persons with ILI who presented for care at two hospitals in Maracay, Venezuela, from October 2006 to December 2010. A respiratory specimen and clinical information were obtained from each participant. Viral isolation and identification with immunofluorescent antibodies and molecular methods were employed to detect respiratory viruses such as adenovirus, influenza A and B, parainfluenza, and respiratory sincytial virus, among others. There were 916 participants in the study (median age: 17 years; range: 1 month – 86 years). Viruses were identified in 143 (15.6%) subjects, and one participant was found to have a co-infection with more than one virus. Influenza viruses, including pandemic H1N1 2009, were the most frequently detected pathogens, accounting for 67.4% (97/144) of the viruses detected. Adenovirus (15/144), parainfluenza virus (13/144), and respiratory syncytial virus (11/144) were also important causes of ILI in this study. Pandemic H1N1 2009 virus became the most commonly isolated influenza virus during its initial appearance in 2009. Two waves of the pandemic were observed: the first which peaked in August 2009 and the second - higher than the preceding - that peaked in October 2009. In 2010, influenza A/H3N2 re-emerged as the most predominant respiratory virus detected.

**Conclusions/Significance:**

Influenza viruses were the most commonly detected viral organisms among patients with acute febrile respiratory illnesses presenting at two hospitals in Maracay, Venezuela. Pandemic H1N1 2009 influenza virus did not completely replace other circulating influenza viruses during its initial appearance in 2009. Seasonal influenza A/H3N2 was the most common influenza virus in the post-pandemic phase.

## Introduction

Acute respiratory infection (ARI) remains a leading cause of global burden of disease, and is the second most common cause of illness worldwide, with an annual global incidence exceeding 400 million [1]–[3]. A prerequisite of public health planning to reduce global disease burden from ARI is to examine data on its epidemiology in order to better define environmental factors as well as target populations for preventive interventions [4]. Respiratory viruses are predominant causes of ARIs, and the epidemiology of acute viral respiratory illnesses in developed countries with temperate climates has been well-characterized [5]–[7]. In countries such as the United States, children have been shown to carry a large burden of viral respiratory diseases [5]. Recent prospective studies, which utilized more sensitive methods for detecting respiratory viruses such as multiplex polymerase chain reaction (PCR), have similarly demonstrated that the highest rates of viral respiratory infection occur among children and the frequency of infection tends to decrease with age due to increasing acquired immunity [8]. Respiratory syncytial virus (RSV), influenza virus, parainfluenza virus, and rhinovirus have long been identified as common causes of ARI [9]. Recent improvements in molecular detection techniques have allowed the identification of multiple new respiratory viruses such as human metapneumovirus (hMPV), human bocavirus (HBoV) and human coronavirus NL63 [8]. While the body of literature describing the epidemiology of acute viral respiratory diseases in developed countries has rapidly expanded, knowledge of the distribution of these diseases in regions such as tropical South America remains limited.

Influenza viruses are among the most impactful acute respiratory pathogens in terms of morbidity and mortality. Despite developed public health intervention programs, the estimated annual average number of influenza-related hospitalizations in the United States exceeds 200,000, and 36,000 deaths are attributable to influenza infections yearly [10], [11]. Information on the contribution of influenza viruses to the global burden of disease due to acute respiratory illness is incomplete. Data on the epidemiology of influenza viruses in developed countries are derived from multiple sources to include laboratory-based surveillance, sentinel surveillance, as well as hospitalization and outpatient records. In developing countries, where resources are sparse, sentinel surveillance methods may be more readily accessible and more cost-effective than laboratory-based or population-based surveillance for determining the viral etiology of influenza-like illness (ILI) in these regions. Better identification of the viral causes of ILI will enable clinicians in resource-limited settings to appropriately treat and manage patients; more importantly, it will allow public health officials to formulate more effective prevention and control strategies, including monitoring of influenza vaccine efficacy in their communities [12].

Studies on the epidemiology of ILI in the tropical South and Central American countries of Peru, Brazil, Ecuador, Nicaragua, Honduras, and El Salvador have been published [13]–[16]. A prospective study of adults with ILI in Sao Paulo, Brazil, revealed that while influenza viruses were the predominant cause of ILI, rhinoviruses and other respiratory viruses were detected in 19.6% and 13.7% of subjects, respectively [14]. This observation illustrated that a significant proportion of patients who are clinically diagnosed with influenza virus infection may have symptoms indistinguishable from other respiratory viruses. In a prospective study of ILI in Ecuador, the regional distribution of influenza virus infections varied; a higher detection rate of influenza A occurred in Quito, located in the highlands where the level of absolute humidity is lower, whereas influenza A detection rate was lower in the coastal city of Guayaquil, which has a more humid tropical climate [15]. Additionally, an expanded sentinel surveillance of ILI conducted at 31 health centers and hospitals located in 13 Peruvian cities showed that the distribution of ILI-causing viruses varied by region [13]. These studies illustrate the importance of conducting baseline and continuing ILI surveillance in other countries of Central and South America because ILI can be caused by pathogens other than influenza viruses. The distribution of respiratory infections may vary within the region or within a particular country, depending on climate and topography.

In Venezuela, ARI is the primary cause of weekly notifiable diseases registered by the National Epidemiological Surveillance System (NESS) [17]. Nonetheless, very low numbers of respiratory samples were collected (16,664 from 2006 to 2010) and even lower numbers (5,167) were confirmed by the National System for Virological Surveillance of ARI [17]–[21]. Furthermore, the epidemiology, clinical characteristics, and viral causes of ILI have been poorly characterized in Venezuela; to our knowledge, only one longitudinal study, carried out during a limited period of time (February 2005–July 2006) and with a low number of patients (n = 102) with ARI, has been published [22]. The objective of this paper is to describe the epidemiology of ILI using data from a sentinel surveillance system at two major hospitals in Maracay, Venezuela.

## Results

### General Findings

A total of 916 subjects participated in this study. Six hundred and thirty seven (69.5%) were recruited at the Hospital Central de Maracay (HCM). There was a slightly larger proportion of females compared with males who enrolled in the study (55.2% vs 44.8%), and the gender distributions at the two sites were similar. Subject ages ranged from 1 month to 86 years with a mean age of 19.2 years (S.D. = 14.3 years) and a median of 17 years. A significant percentage of the subjects (17.6%) were under 5 years of age, while less than 1.0% were 60 years or older. The 15–29 year old group was the most predominant group comprising 32.8% of the study population, followed by the 5–14 year old age group (27.2%). The subjects who presented to HCM were significantly older (mean age = 21.2 years; S.D. = 13.2 years) compared with those who were seen at the Hospital del Instituto Venezolano de los Seguros Sociales Jose Maria Carabano Tosta (IVSS-JMCT) (mean age = 14.4 years; S.D. = 15.6 years). A large proportion (37.8%) of the subjects at HCM belonged to the 15–29 age group, while nearly half of the patients (42.3%) seen at IVSS-JMCT were younger than 5 years old. Approximately 5% of the subjects reported having received influenza vaccination within the last year. A small percentage of patients (2.0%) received antibiotic treatment for their acute respiratory illness prior to enrollment in the study. No hospitalized cases with ILI were recruited in this study; only outpatient subjects participated. Demographic information is summarized in Table 1.

**Table 1:** Study population recruited by passive surveillance in two hospitals of Maracay, Venezuela: October 2006– December 2010.

Characteristics of the population	HCM*	IVSS†		Total
	No. (%)	No. (%)		No. (%)
**Number of subjects enrolled**	637	279		916
**Gender**				
Female	344 (54.0)	162 (58.1)		506 (55.2)
Male	293 (46.0)	117 (41.9)		410 (44.8)
**Age**				
Mean, ±STD (yrs)	21.2±13.2	14.4±15.6		19.2. ±14.3
Median, (range in yrs)	20 (1 mo–86)	7 (1 mo–65)		17 (1 mo –86)
0–4	43 (6.8)	118 (42.3)		161 (17.6)
5–14	196 (30.8)	53 (19.0)		249 (27.2)
15–29	241 (37.8)	59 (21.1)		300 (32.8)
30–44	126 (19.8)	30 (10.8)		156 (17.0)
45–59	25 (3.9)	18 (6.5)		43 (4.7)
≥60	6 (0.9)	1 (0.4)		7 (0.8)
**Influenza vaccination (self-reported)**	31 (4.9)	17 (6.1)		48 (5.2)
**Medical attention before enrollment**	175 (27.5)	29 (10.4)		204 (22.3)
**Previous treatment**				
Treatment	14 (2.2)	21 (7.5)		35 (3.8)
Including antibiotics	9 (1.4)	9 (3.2)		18 (2.0)
No treatment	468 (73.5)	168 (60.2)		636 (69.4)
No information	155 (24.3)	90 (32.3)		245 (26.7)

* Hospital Central de Maracay.

† Hospital del Instituto Venezolano de los Seguros Sociales-José María Carabaño Tosta.

### Laboratory Results

Among the 916 patient samples obtained from the sentinel surveillance, at least one viral organism was detected by viral isolation and/or reverse transcriptase-polymerase chain reaction (RT-PCR) in 143 (15.6%) subjects (Table 2). Only one participant with a co-infection was observed. Influenza viruses were the most frequently detected organisms in this passive surveillance, consisting of 67.4% (97/144) of all the viruses detected by viral isolation and/or RT-PCR. Of the 916 samples collected, 78 (8.5%) were positive for influenza A viruses and 19 (2.1%) for influenza B viruses by viral isolation and/or RT-PCR. In addition, non-influenza viruses were detected only by virus isolation; they were: adenovirus (1.6%), parainfluenza virus (1.4%), RSV (1.2%), and other viruses (0.8% ), which included HBoV (0.1%), enterovirus (0.2%), hMPV (0.1%), rhinovirus (0.1%), and herpes simplex virus (HSV, 0.2%). The only co-infection was observed in a 22 month old toddler in whom adenovirus and HSV were isolated.

**Table 2:** Age distribution of febrile respiratory viral infections detected by passive surveillance in two Hospitals of Maracay, Venezuela: October 2006–December 2010.

Age group (in years)								
	0–4	5–14	15–29	30–44	45–59	≥60		Total
Viral Pathogen	No. (%)	No. (%)	No. (%)	No. (%)	No. (%)	No. (%)		No. (%)
**Adenovirus**	9*(5.6)	3 (1.2)	0	2 (1.3)	1 (2.3)		0	15 (1.6)
**Influenza virus A:**								
** H3** †	9 (5.6)	8 (3.2)	7 (2.3)	8 (5.1)	0		1 (14.3)	33 (3.6)
** H1** ‡	3 (1.9)	2 (0.8)	1 (0.3)	0	0		0	6 (0.7)
** Seasonal not subtyped**	3 (1.9)	2 (0.8)	4 (1.3)	1 (0.6)	1 (2.3)		0	11 (1.2)
** pH1N1** §	2 (1.2)	3 (1.2)	13 (4.3)	3 (1.9)	0		0	21 (2.3)
** Not subtyped**||	1 (0.6)	2 (0.8)	4 (1.3)	0	0		0	7 (0.8)
**Influenza virus B**	4 (2.5)	10 (4.0)	5 (1.7)	0	0		0	19 (2.1)
**Parainfluenza virus**	6 (3.7)	1 (0.4)	3 (1.0)	3 (1.9)	0		0	13 (1.4)
**RSV** ¶	8 (5.0)	0	1 (0.3)	2 (1.3)	0		0	11 (1.2)
**ORV** **	2 (1.2)	2 (0.8)	0	3 (1.9)	0		0	7 (0.8)
**Positives**	47 (29.2)	33 (13.3)	38 (12.7)	22 (14.1)	2 (4.7)		1 (14.3)	143 (15.6)
**Negatives**	114 (70.9)	216 (86.7)	262 (87.3)	134 (85.9)	41 (95.3)		6 (85.7)	773 (84.4)
**Total**	161	249	300	156	43		7	916

* One mixed infection with herpes simplex virus in a 22 month old toddler.

† Influenza A/H3 subtype.

‡ Influenza A/H1 subtype (non-pandemic).

§ pH1N1: Pandemic (H1N1) 2009 influenza virus.

|| Influenza A isolated but not subtyped by RT-PCR (unknown subtype).

¶ Respiratory syncytial virus.

** ORV: Other respiratory viruses, which includes human metapneumovirus, human bocavirus, herpes simplex virus, and enterovirus.

The virus etiology and detection rates varied with age (Table 2). The most commonly detected viral pathogens in the 0–4 year old group were influenza A (11.1%) and adenoviruses (5.6%). Influenza A and B were the most commonly detected viruses in the school-age group (5–14 year old: 6.8% and 4.0%, respectively) and the late adolescent and young adult group (15–29 year-old: 9.7% and 1.7%, respectively). Influenza A was the only detectable virus among patients who were 60 years or older.

Of the 78 influenza A cases, H3 subtype was detected in 33, and was observed in all the age groups but in subjects aged 45–59 year old (Table 2). Six cases had the H1 subtype (non-pandemic), and 18 influenza A viruses were isolated but not subtyped by one-step RT-PCR; of the latter, 11 can be classified as seasonal because they were isolated from patients samples collected before the start of the H1N1 pandemic outbreak of 2009. Twenty-one cases of pandemic H1N1 2009 influenza virus (pH1N1) infections were identified in this sentinel surveillance; thirteen occurred in the 15–29 year old group. There were no cases of pH1N1 infections among adults 45 years old or higher.

### Phylogenetic Analysis

**Figure 1:**
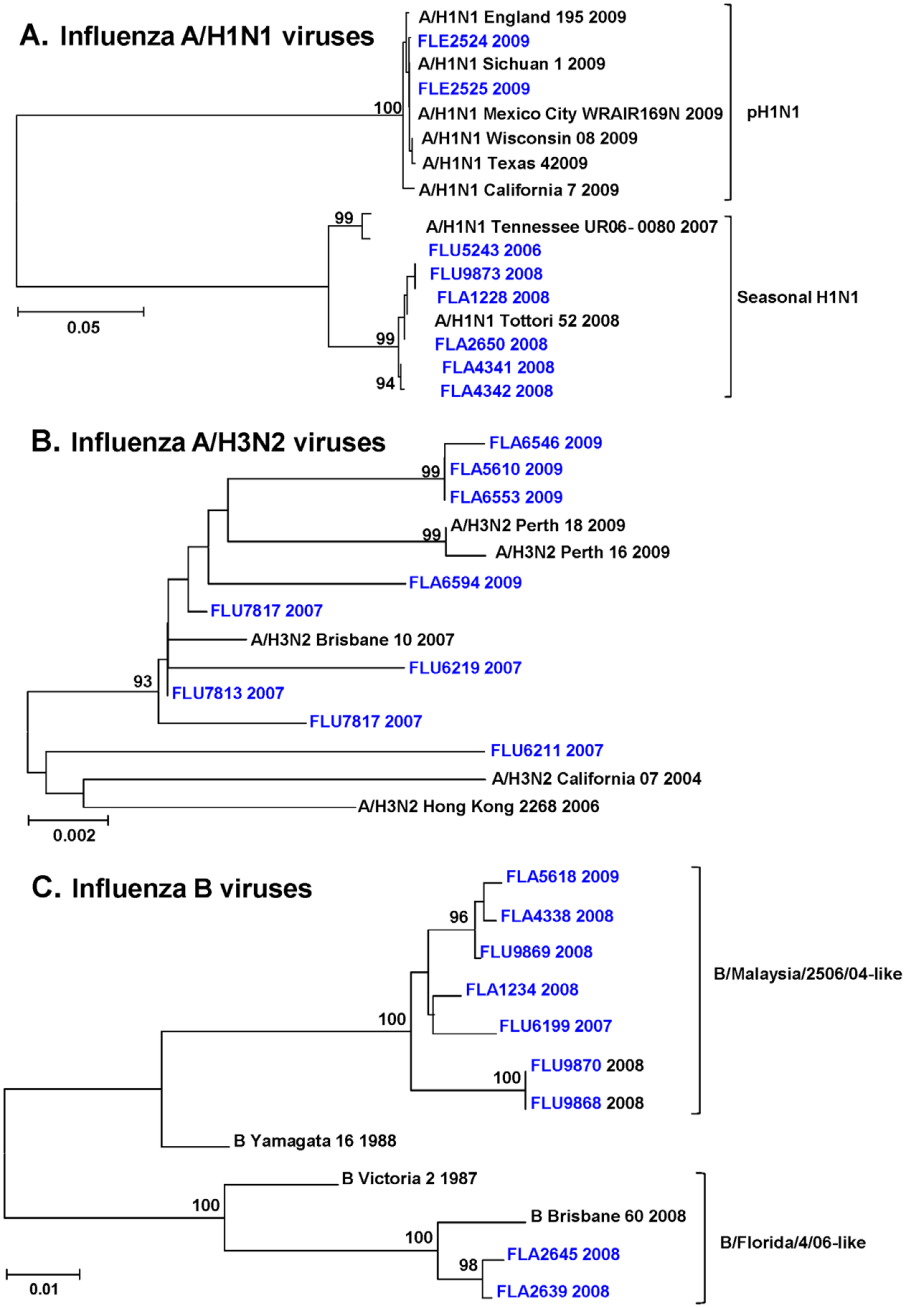
Phylogenetic trees of influenza viruses circulating in Maracay. This figure shows the phylogenetic relationship of the HA gene segment within influenza A/H1N1 (A), influenza A/H3N2 (B) and influenza B (C) viruses. Phylogenetic trees were constructed by the neighbor-joining method and bootstrap analysis to determine the best-fitting tree for the gene. For the comparison, we have included strains reported from GenBank. Only bootstrap values over 90% are shown.

Genetic analysis based on partial hemagglutinin gene sequences (approximately 900 bp) of 27 influenza isolates is shown in Figure 1 (See Material and Methods for GenBank accession numbers). This analysis shows that the circulating seasonal A/H1N1 strains in Venezuela (Figure 1A) were similar to the ones previously described in Central and South America [15], [16] and all of these samples can be grouped with the A/Brisbane/56/07 like 2008–2009 genotype. The pH1N1 samples were part of only one cluster similar to previously reported strains in Latin America [23]. Influenza A/H3N2 samples possessed more genetic variability (Figure 1B); isolates from 2007 revealed two genotypes, A/Brisbane/10/07-like and A/California/7/04-like, while the more recent isolates were closer to the A/Perth/16/09-like genotype. Finally, in Figure 1C, the genetic analysis for influenza B isolates disclosed the presence of two different genotypes, B/Florida4/06-like and B/Malaysia/2506/07-like, in agreement to what was previously found in the region [13], [16].

### Temporal Distribution

**Figure 2:**
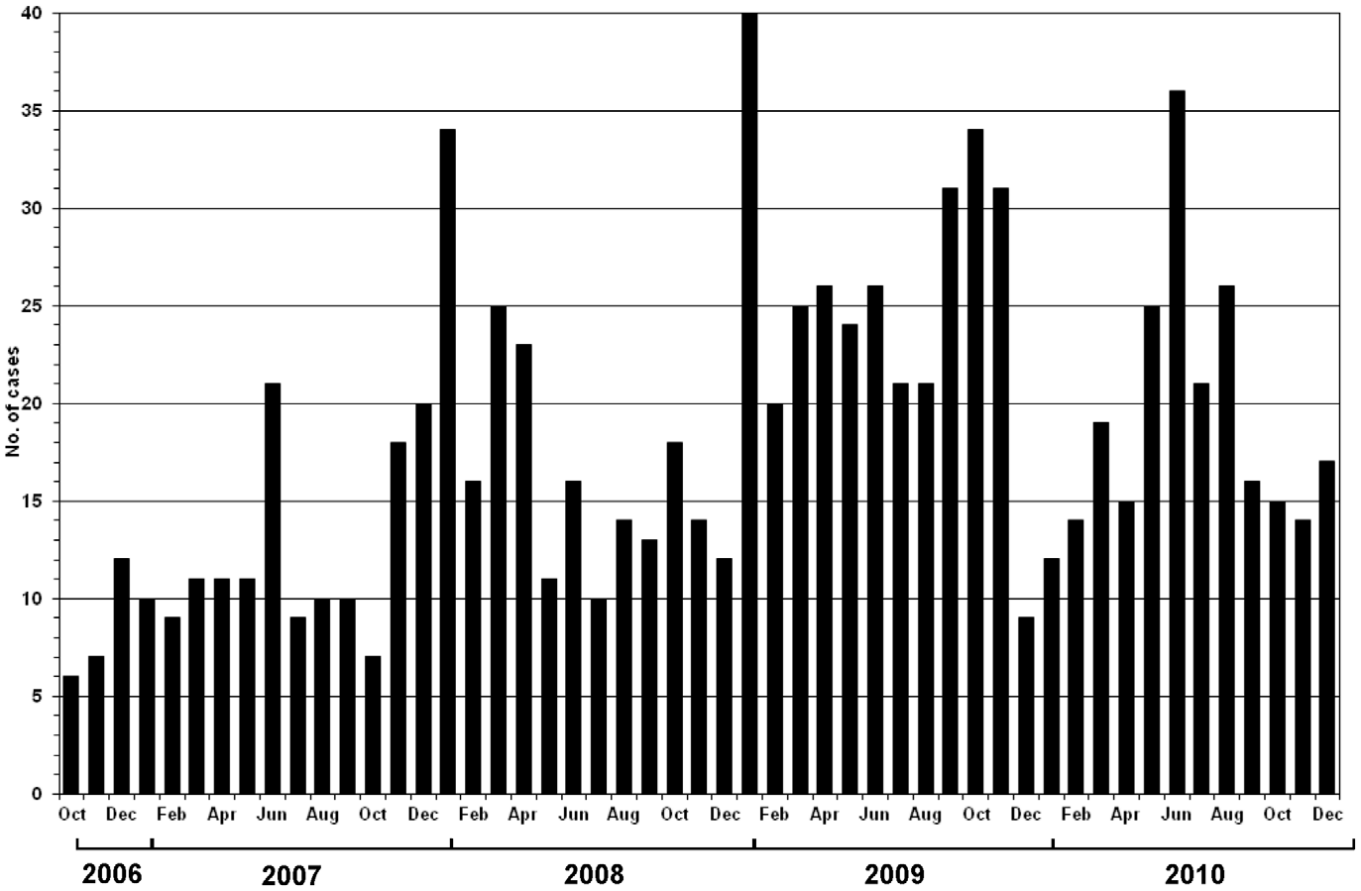
Influenza-like illness detected by passive surveillance in Maracay, Venezuela: October 2006–December 2010.

**Figure 3:**
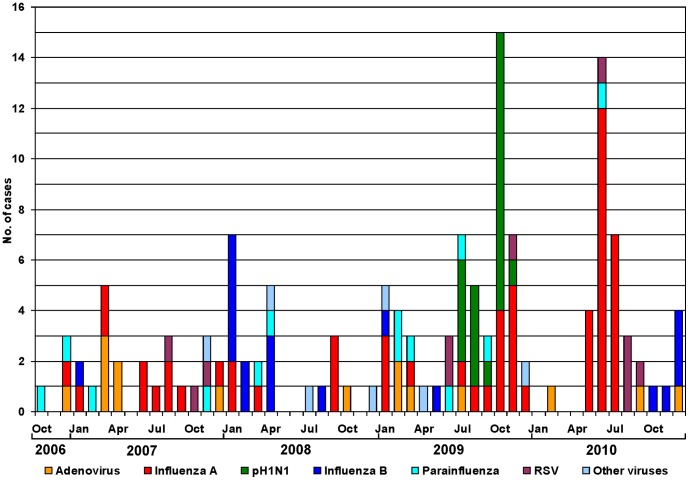
Monthly distribution of acute febrile respiratory viral infections by different viruses detected through a passive surveillance in two health centers of Maracay.

Figures 2 and 3 illustrate the temporal distribution of ILI and the viral etiology of ILI, respectively, from October 2006 through December 2010. ILI was observed throughout the year with irregular peak activity occurring once or twice annually during the months of June 2007, January 2008, January and October 2009, and June 2010 (Figure 2). The pattern of influenza A occurrence was variable from year to year (Figure 3). Influenza A virus was not detected during every month of every year and was observed during 3–6 month periods in 2007, 2009, and 2010 (4, 6, and 3 months, respectively). Lower influenza A activity was observed in 2008, with detection only during the months of January, March, and September. The occurrence of peak ILI activity correlated with the detection of influenza A virus in the subjects’ respiratory samples during those months (see Figures 2 and 3). Peak influenza B virus activity was detected in January 2008 and December 2010. Adenovirus was detected more frequently from December to April. RSV infection occurred more frequently from June to November. Parainfluenza viruses were detectable throughout the year but without distinct seasonality.

The passive surveillance illustrated the impact of pH1N1 on the distribution of respiratory viruses associated with ILI in 2009 (Figure 3). During the ILI peaks from 2006–2010, the percentage of ILI cases attributable to influenza viruses ranged from 10% to 44%. The first cases of pH1N1 in this surveillance system were detected in July 2009, three months after the swine origin influenza outbreak began in Mexico [24]. During the first wave in July 2009, the monthly virus detection rate rose to 33%, and 57% (4/7) of all the viruses detected were of the pandemic strain. The first wave simulated a typical ILI peak activity similar to those observed in other years. However, during the larger second wave in October 2009, the virus positivity rate exceeded 40%, and the pandemic strain was identified in greater than 73% (11/15) of the viruses detected. Figure 3 demonstrates that pH1N1 did not completely replace seasonal influenza A viruses. The peak pandemic activity in October 2009 was followed by a rapid decline in the rate of pandemic strain detection one month later. Meanwhile, seasonal influenza A viruses remained in circulation throughout the pandemic period comprising 27% of all the influenza A viruses detected in October 2009. In 2010, 20% of influenza A specimens obtained via oropharyngeal swabs were randomly selected and tested for the pandemic strain via RT-PCR, and no cases of pH1N1 were detected (Figure 3). However, seasonal influenza A/H3N2 continued to be detected by our surveillance system with monthly positivity rates ranging from 16% to 30%.

### Clinical Manifestations

The clinical features of subjects in our surveillance are summarized in Table 3. Compared with those whose respiratory secretions tested negative, subjects in whom virus was identified were more likely to have sore throat, headache, pharyngeal congestion, and ear pain. There were no significant differences in the symptoms of individuals who had seasonal influenza A when compared with those who suffered from pH1N1 influenza, except that a higher proportion of the latter subjects had expectoration, myalgias, and lymphadenopathy. The symptoms of influenza A (either seasonal or pandemic) and influenza B were clinically indistinguishable. When compared with patients who had ILI due to other viruses, a higher percentage of those with confirmed influenza virus infection experienced sore throat, myalgias, and headache.

**Table 3:** Signs and symptoms of patients with acute febrile respiratory infections detected by virus identified in two health centers of Maracay, Venezuela: October 2006–December 2010.

	Total	No Vírus Detected	Virus Detected	Seasonal Influenza A	Influenza B	p(H1N1)	Other viruses**
	N = 916	N = 773	N = 143	N = 50	N = 19	N = 21	N = 46
Signs/Symptoms	No. (%)	No. (%)	No. (%)	No. (%)	No. (%)	No. (%)	No. (%)
**Cough**	880 (96.1)	749 (96.9)	131 (91.6)	46 (92.0)	18 (94.7)	20 (95.2)	40 (87.0)
**Malaise**	864 (94.3)	730 (94.4)	134 (93.7)	45 (90.0)	18 (94.7)	21 (100.0)	43 (93.5)
**Rhinorrhea**	861 (94.0)	729 (94.3)	132 (92.3)	48 (96.0)	16 (84.2)	17 (81.0)	42 (91.3)
**Sore throat**	178 (19.4)	135 (17.5)	43 (30.1)*	21 (42.0)	4 (21.1)	9 (42.9)	8 (17.4)‡
**Expectoration**	536 (58.5)	459 (59.4)	77 (53.8)	23 (46.0)	7 (36.8)	18 (85.7)†	23 (50.0)
**Myalgias**	499 (54.5)	433 (56.0)*	66 (46.2)	20 (40.0)	8 (42.1)	16 (76.2)†	15 (32.6)‡
**Headache**	388 (42.4)	309 (40.0)	79 (55.2)*	30 (60.0)	9 (47.4)	15 (71.4)	19 (41.3)‡
**Wheezing**	342 (37.3)	310 (40.1)*	32 (22.4)	9 (18.0)	6 (31.6)	3 (14.3)	14 (30.4)
**Shortness of breath**	267 (29.1)	224 (29.0)	43 (30.1)	12 (24.0)	9 (47.4)	9 (42.9)	12 (26.1)
**Pharyngeal congestion**	132 (14.4)	81 (10.5)	51 (35.7)*	21 (42.0)	4 (21.1)	6 (28.6)	15 (32.6)
**Asthenia**	71 (7.8)	59 (7.6)	12 (8.4)	3 (6.0)	3 (15.8)	2 (9.5)	2 (4.4)
**Ear pain**	55 (6.0)	37 (4.8)	18 (12.6)*	8 (16.0)	0	2 (9.5)	3 (6.5)
**Diarrhea**	53 (5.8)	44 (5.7)	9 (6.3)	2 (4.0)	1 (5.3)	2 (9.5)	3 (6.5)
**Conjunctival injection**	36 (3.9)	18 (2.3)	18 (12.6)	7 (14.0)	2 (10.5)	2 (9.5)	5 (10.9)
**Lymphadenopathy**	18 (2.0)	12 (1.6)	6 (4.2)	0	0	3 (14.3)†	1 (2.2)
**Abdominal pain**	14 (1.5)	9 (1.2)	5 (3.5)	3 (6.0)	0	2 (9.5)	0

* Statistically significant differences (p<0.05) between patients with ILI in whom virus was detected vs. virus was not detected.

† Statistically significant differences (p<0.05) between patients with seasonal influenza A and pH1N1.

‡ Statistically significant differences (p<0.05) between patients with ILI – due to influenza viruses and ILI – due to viruses other than influenza viruses.

** Other viruses: human metapneumovirus, human bocavirus, herpes simplex virus, respiratory syncytial virus, adenovirus, parainfluenza virus, and enterovirus.

## Discussion

There is scarce information about the epidemiology of acute febrile respiratory illness in Venezuela and, to our knowledge, only one longitudinal study has been published [22]. This investigation, however, was limited to few number of subjects (n = 102) recruited during a short period of time (17 months) and did not describe the transmission seasonality of the viral infections. Thus, our study is the first to fully describe the epidemiology and viral etiology of ILI in Venezuela and provides baseline levels of ILI activity in a typical highly-populated urban city.

Our study demonstrated that influenza viruses are a main cause of ILI at HCM and IVSS in Maracay in agreement with findings reported by Venezuelás NESS [17]–[21]. Nevertheless, the rate of confirmed influenza virus infections found in our surveillance (10.6%, 97/916; Tables 2 and 3) during the study period was lower than the one (27.7%) reported by the NESS for the same period [17]–[21]. These differences in detection rates may be attributable to different strategies for capturing ARI patients, especially those with influenza, used by the NESS and by this study protocol (Flores E, Director of Epidemiology, Corposalud 2011, personal communication). The NESS randomly selected a sample of ARI cases (including those with influenza) with emphasis on severe hospitalized cases, whereas in our protocol we recruited ILI subjects in an outpatient setting where the majority had symptoms that were not severe. On the other hand, the percentage of influenza viruses (not including pH1N1) detected in our study during a similar period of time, but in different years (February 2007– July 2008: 22 of 38, 57.9%; data not shown), was much higher than the one reported by Valero et al. (February 2005– July 2006: 7 of 46, 15.2% ) [22]. Two causes may have accounted for the significant differences found in both studies: a) the collection, preservation and further processing of respiratory samples, and b) the type of cells and IFA reagents used for virus isolation and identification.

The proportion of influenza cases was significantly different when comparing non-pandemic and pandemic periods. Before the H1N1 2009 pandemic, the NESS [21] detected influenza virus in 64.7% of subjects in whom a virus was isolated; a similar proportion to the 55% (data not shown) found in our study. During the height of the pandemic (from July through Dec 2009), the NESS confirmed 97% of all ARI cases with a virus as having either influenza A or B virus compared with 87% observed in our study.

The predominance of influenza viruses as etiological agents of ILI in Maracay, Venezuela, is consistent with observations of surveillance studies in other tropical Central and South American countries [13], [15], [16]. While the overall virus detection rate (143/916, 15.6%; Table 2) was lower compared with other studies, the positive rates for influenza A (8.5%) and influenza B (2.1%) in this surveillance were comparable to those observed in a similar study in El Salvador, Honduras, and Nicaragua [16]. Our findings were also consistent with a study in Indonesia, which identified influenza A or B in 11% of all respiratory samples using viral isolation and RT-PCR [25]. Our findings were consistent with those from a prospective study of outpatient children in northern Taiwan, in which influenza A or B were isolated in 12.2% of subjects with ILI, using Madin-Darby canine kidney (MDCK) cell cultures and hemagglutinin inhibition assay for antigen detection [26]. However, our detection rate for all respiratory viruses, as well as influenza A and B viruses, was generally lower compared to other studies conducted in South America. In an expanded sentinel surveillance study in Peru, which utilized a similar methodology for viral detection as our study, the virus positivity rate was 42.1%, and influenza A and B rates were 25.1% and 9.7%, respectively [13]. In a prospective surveillance study of ILI in two Ecuadorian cities using similar methods, at least one virus was detected in 35% of the participants; influenza A was detected by PCR in 21.6% while 6.4% tested positive for influenza B [15]. The lower virus detection rates found in our study is unclear because we used the same operative protocol described in the mentioned studies [13], [15]. Nevertheless, the different skills to collect respiratory tract specimens from ILI patients, by the health personnel employed in their studies and ours may have accounted for the lower virus detection rates found in our study.

While influenza viruses were observed to be the most prevalent viral pathogen, adenovirus, parainfluenza virus, and RSV were important causes of ILI at the two hospitals (Table 2). In Venezuela, the NESS reported RSV as the most frequently detected non-influenza respiratory virus followed by parainfluenza virus and rhinovirus [17]–[21]. The study performed in Zulia, Venezuela, also reported RSV as the most common detected virus, followed by adenovirus, parainfluenza virus and influenza viruses [22]. As previously mentioned, the differences in the detection rates may be attributed to the different procedures used by the NESS, other Venezuelan researchers. [22] and us. These viral pathogens were also frequently detected in other surveillance studies in Central and South America [14]–[16].

The highest detection rate of respiratory viruses was observed in the 0–4 year old group (29.2%; Table 2). The rate of viral positivity generally decreased with age as acquired immunity increased in older subjects. A slight increase can be seen between the 30–44 year old group (14.1%) and 60 years or older group (14.3%). Subjects who were 60 years old or older comprised less than 1% of the study population, and thus, the prevalence in this age group may be unreliable. Contrary to our study, the other study from. Venezuela had higher detection rates in both the 0–6 and ≥41 year old groups (48.2% and 57.1%, respectively) [22]. On the other hand, our findings are consistent with observations in the Tecumseh Study, a community-based surveillance in Michigan, in which children were shown to have the highest rate of viral respiratory diseases [5], [6]. The Tecumseh study further illustrated the variable impact of influenza viruses among the different age groups depending on the influenza virus subtype. For example, influenza A/H3N2 affected a wide range of age groups while influenza A/H1N1 and influenza B virus infections occurred more frequently among older children and young adults [5], [6]. Our findings were similar being seasonal influenza A/H1N1 viruses and influenza B detected primarily in children and young adults, and seasonal influenza A/H3N2 found in all age groups.

As expected, pH1N1 was the most common influenza A subtype identified among the subjects with ILI in 2009 (Figure 3). In 2009, 37.5% (Figure 3) of ILI cases were due to pH1N1; this detection rate was lower than the 52.2% detection rate reported by the Venezuela’s NESS [21]. Nevertheless, this finding was similarly demonstrated in respiratory illness surveillance networks in other tropical and temperate South American countries such as Guatemala [27], Peru [28], Argentina [29], and Brazil [30]. In 2010, non-pandemic influenza viruses continued to circulate in Venezuela, and pH1N1 was not detected in our surveillance study, suggesting that pH1N1 did not displace seasonal influenza A viruses. During the same year, 1.6% of the ARI cases reported to Venezuela’s NESS were due to pH1N1 [21]. Infection with pH1N1 may have stimulated immunity among the residents of this community during its initial arrival in 2009. This observation suggests that the circulating pH1N1 in 2010 may not have significantly mutated relative to the strain in 2009, so that antibodies stimulated by the natural infection during its initial arrival may have still been highly efficient in protecting the community from another wave of pH1N1 outbreak. In 2010, the infection rate of influenza A/H3 was higher than the rates observed during previous years, further illustrating the lack of cross protection between pH1N1 and influenza A/H3.

Our study shows that hMPV and HBoV were not commonly associated with ILI, based on the low detection rates observed in our surveillance. In a study from Ecuador which utilized methods similar to those employed in our study, a low detection rate for HBoV (0.2%) was reported [15]. The latter study, as well as another from Central America which used methods similar to ours, reported rare detection of hMPV (<0.2%) [15], [16]. In contrast, a prospective study of ILI among Brazilian adults, which utilized viral isolation and RT-PCR testing on respiratory samples, detected rhinoviruses in 19.6% of patients [14]. Although rhinoviruses are typically associated with milder illness, they can contribute to the misdiagnosis of influenza based on clinical case definition alone. A cohort study of Vietnamese children hospitalized for acute febrile respiratory illness, which applied multiplex-PCR assays on respiratory samples, revealed slightly higher prevalence rates of HBoV (2%) and hMPV (5%) infections [31]. It is important to note that our method of identification (culture on three cell lines), compared to molecular diagnostic methods, substantially lacked sensitivity for detecting rhinoviruses, hMPVs, and HBoVs.

Our study shows that patients with pH1N1 infections were more likely to have myalgias, productive cough with expectoration, and lymphadenopathy than with those infected with seasonal influenza A virus (Table 3). In contrast, clinical manifestations in Guatemalan subjects hospitalized for pH1N1 and seasonal influenza A infections did not significantly differ [27].

Our study had limitations worth noting. Data collection at only two hospitals in an urban area limits our ability to generalize our findings to the population. The low virus detection rates may be attributable to variations in the skills of the health staff employed to collect the respiratory specimens. The sampling method may have a significant effect on the proportion of respiratory viruses identified, and nasopharyngeal washes may yield higher sensitivity over nasopharyngeal or oropharyngeal swabs [32]. Study participants were exclusively seen in the outpatient setting, thus limiting our ability to examine the impact of respiratory viruses in hospitalized cases. A large proportion of ILI cases were not associated with any pathogen, and the impact of bacteria on this clinical syndrome cannot be determined from this study, since the respiratory samples were not cultured for bacteria. Two methods of detection were used for identification of influenza (PCR and culture) whereas only one method of detection was used for the other viruses (culture). Twenty-two percent (9/40) of the respiratory samples which were positive for influenza viruses by RT-PCR were negative by viral isolation illustrating that viral detection by culture underestimated the true prevalence. Viral culture may not be the ideal way of isolating organisms such as RSV, hMPV, HBoV and rhinoviruses leading to significant underestimation of their detection rates [33]. Despite these limitations, our study contributes information on the distribution and etiology of ILI at two hospitals in Maracay, Venezuela. This knowledge can serve as a baseline for future, more expansive population-based surveillance studies of influenza and other respiratory viruses in this region.

## Materials and Methods

### Study Sites

**Figure 4:**
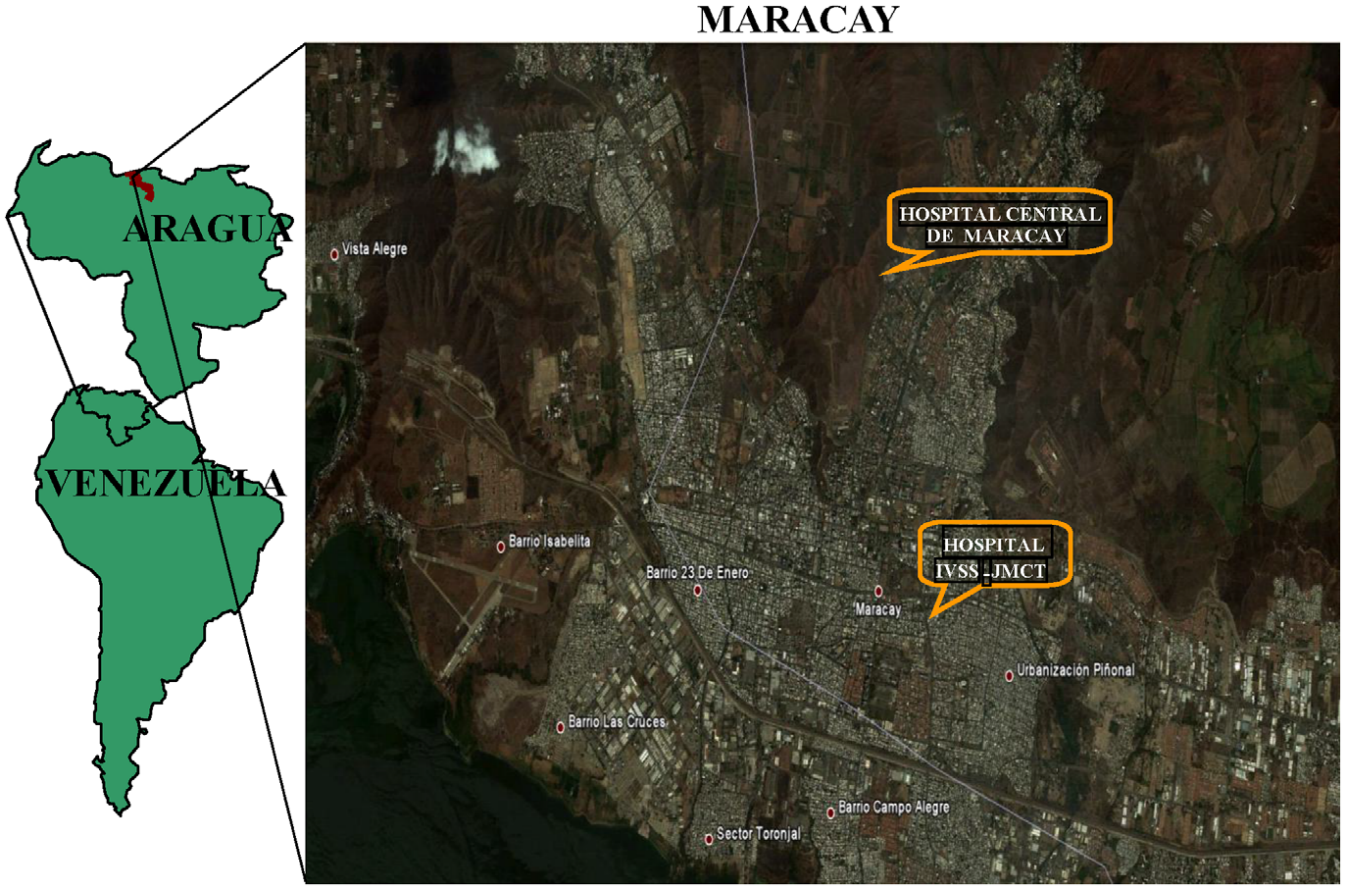
Map of study sites in Venezuela.

Maracay is located in the central northern region of Venezuela (10° 15′ N, 67° 39′ W), approximately 27 miles from the Caribbean coast (Figure 4). The climate is tropical with two seasons: dry (December–April) and rainy (May–November). The monthly averages for temperature, relative humidity and rainfall are 25.1°C (range = 23.4°C–27.6°C), 75% (range = 66%–82%) and 56 mm (range: 0 mm–187 mm), respectively. Maracay is comprised of two main urban municipalities, named Girardot and Mario Briceño Iragorry, and had an estimated total population of 677,359 in 2010. The HCM and the IVSS-JMCT are located approximately 4 miles apart. Both are major county hospitals and referral health centers with adult and pediatric departments including emergency services and 241 (IVSS-JMCT) to 433 (HCM) hospitalization beds. They provide services to people in Maracay as well as those from the adjacent city of Aragua and three neighboring states.

### Study Population

The study population included every outpatient with ILI, regardless of age, who sought attention at the two study sites between October 2006 and December 2010, and agreed to participate in the study. At each site, trained medical personnel were responsible for properly identifying and classifying patients with ILI.

### Case Definition

Each person with ILI was asked to enroll in the study. A person was defined as having ILI if he or she had a sudden onset of fever (≥38°C) and either cough or sore throat for less than five days in duration, with or without general symptoms such as myalgias, prostration, headache, or malaise [13].

### Data Collection and Management

Data on gender, age, previous treatments, medical attention before enrollment, influenza vaccination status, and days of work/school lost at the time of acute illness were collected utilizing a case report form (CRF) from all participants who met the case definition criteria. Temporal distribution of the results were recorded by month during the study period, taking into account the number of ILI cases identified and the number of confirmed cases of influenza A and B in each study site. Monthly reports of enrolled ILI participants and laboratory results were sent to the Venezuelan Ministry of Health. Regular personnel training in protocol procedures were conducted as part of the strategy to improve sampling, storage,and shipping procedures.

### Ethical Management

This protocol was approved as less than minimal risk research by the Naval Medical Research Center (NMRC), Silver Spring, Maryland. Institutional Review Board (IRB; Protocol NMRCD.2002.0019) authorization was given to perform the study using an information sheet approved and stamped by the IRB. As this was part of clinical care and routine surveillance benefiting the ministry of health, verbal consent was obtained from all participants. This method of consent was accepted by the NMRC IRB as well as the Venezuelan institutions involved. A verbal consent was approved by both IRBs following CIOMS and 45CFR46 (the Common Rule), 1) it was a minimal risk study, and 2) local health installations would not require a written consent for the procedures required in this study. Additionally, this document included all the information that a written consent would require; a copy was provided to each study subject; and study personnel responsible for administering the process of informed consent were trained at each site on human research protection issues and were certified through the Collaborative Institutional Training Initiative (CITI). Finally, the frequent monitoring visits conducted by the study team found no problems in the process nor complaints from study participants.

### Laboratory Analysis

#### Sample collection

Nasal (807 of 916, 88.1%) or oropharyngeal (109 of 916, 11.9%) swabs were obtained from each subject for viral isolation and identification. The swabs were placed in viral transport media and stored at –70°C until they were delivered on dry ice to NAMRU-6 in Lima, Perú, for laboratory analysis. Duplicate swabs were processed and analyzed at the Laboratorio Regional de Diagnostico e Investigacion del Dengue y otras Enfermedades Virales/Instituto de Investigaciones Biomedicas de la Universidad de Carabobo (LARDIDEV/BIOMED-UC) in Maracay, Venezuela, following the same operative protocols used in NAMRU-6.

#### Virus isolation and identification

Nine-hundred and sixteen nasal or oropharyngeal swabs were processed for viral isolation and identification following the procedure described by Laguna-Torres et al. [13]. Briefly, patient specimens were inoculated onto four cell lines: Madin-Darby canine kidney (MDCK; ATCC® Number CCL-34), African green monkey kidney (Vero76; ATCC® NumberCRL-1587) and VeroE6 ATCC® Number CRL-1586), and Rhesus monkey kidney (LLC-MK2 ATCC® Number CCL-7). Upon the appearance of cytopathic effect or after ten days of culture (or thirteen days in the case of Vero cells), the cells were spotted onto microscope slides. Cell suspensions were dried and fixed in chilled acetone for 15 minutes. Virus isolates were identified using direct fluorescence antibody (DFA) assays. The Respiratory Virus Screening and Identification Kit (D3 DFA Respiratory Virus Diagnostic Hybrids; Athens, OH) was utilized for the identification of adenoviruses, influenza A virus, influenza B virus, parainfluenza viruses (types 1, 2, and 3), and RSV. The D3 DFA Herpes Simplex Virus (HSV) identification kit and the D3 IFA Enterovirus ID kit (Diagnostic Hybrids; Athens, OH) were utilized for the identification of HSV (both HSV-1 and HSV-2) and enteroviruses, respectively. For isolation of hMPV, we used Vero E6 and LLC-MK2 cell lines. For detection of hMPV antigens by direct fluorescence assay, we used an anti-hMPV mouse monoclonal antibody from Diagnostic Hybrid (Athens, OH). All assays were performed following the manufacturers’ instructions. HBoV was identified using the methods described by Salmon-Mulanovich, et al [34]. Since duplicate swab samples were analyzed in different laboratories (LARDIDEV/BIOMED-UC and NAMRU-6) with the same diagnostic kit and standard operating procedures, a virus isolation result was considered positive if the specific virus was isolated and identified at either site.

A subset of 254 specimens (222 nasals and 32 oropharyngeals) was analyzed by one-step RT-PCR and/or Real Time RT-PCR in order to sub-type influenza A/H1N1 (including pH1N1), influenza A/H3N2, and influenza B viruses. One-step RT-PCR and/or Real Time RT-PCR were not used to identify non-influenza viruses because the specific primers and protocols were available only for sub-typing influenza viruses; thus, only virus isolation was used to detect and identify non-influenza viruses. Of the 254 specimens, 48 (18.9%) were randomly selected from repository samples collected before the 2009 pandemic and tested for influenza A sub-typing by one-step RT-PCR and/or Real Time RT-PCR. At the onset of the 2009 pandemic, 150 of 254 (59.1%) respiratory samples were tested for pH1N1 by Real Time RT-PCR at the request of the Venezuelan Ministry of Health. After the peak of the second wave of the pandemic, the percentage of respiratory samples tested by Real Time RT-PCR was reduced to 22% (56 of 254).

One-step RT-PCR was performed according the procedure and influenza primers described below. Real Time RT-PCR was carried out using procedures (CDC protocols CDC REF.# I-007-05 Version 2007, CDC REF.# LB-013, R-1 and CDC REF.# I-007-05 Version 2009: Swine Influenza) and materials provided by the Influenza Division of the Centers for Disease Control and Prevention, U.S.A. (Stephen Lindstrom,personal communication). These protocols are available from CDC upon request. For the purpose of this study, an ILI case with a confirmed viral respiratory infection was one in which viral isolation and/or RT-PCR identified a virus.

#### RNA extraction and one-step RT-PCR

Viral RNA extraction was performed from the supernatant of infected MDCK cells using a QIAamp Viral RNA kit (QIAGEN; Valencia, CA) following the manufacturer’s protocol. The one-step RT-PCR was performed following a procedure described previously [13] with primers that amplified the hemagglutinin (HA) gene of influenza A and influenza B viruses using the SuperScript III One-Step RT-PCR System kit (Invitrogen; San Diego, CA). The following primers were used for the amplification of H1 influenza A viruses: H1F-6 (5′-AAGCAGGGGAAAATAAAA-3′) and H1R-1193 (5′-GTAATCCCGTTAATGGCA-3′); for H3 influenza A viruses: H3F-7 (5′-ACTATCATTGCTTTGAGC-3′) and H3R-1184 (5′-ATGGCTGCTTGAGTGCTT-3′); for influenza B viruses: BHAF-36 (5′-GAAGGCAATAATTGTACT-3′) and BHAR-1140 (5′-ACCAGCAATAGCTCCGAA-3′). Five µl of the extracted RNA was added to 20 µl of master mix containing the enzyme mixture (SuperScript III RT/Platinum Taq), 2X reaction mixture (containing 0.4 mM of each dNTP and 3.2 mM of Mg_2_SO4) and 20 µM of each primer. Cycling conditions included a reverse transcription step at 50°C for 30 minutes and a denaturation step at 94°C for 2 minutes. Cycling conditions of the PCR were 40 cycles of 94°C for 15 seconds, 52°C for 30 seconds, and 68°C for 75 seconds, followed by a final incubation step at 68°C for 5 minutes.

#### DNA sequencing and phylogenetic analysis

For confirmation of serotype and genotype of the circulating influenza viruses, 27 samples were sequenced. As part of the Respiratory Surveillance Protocol in Venezuela, these 27 viruses were randomly selected from approximately 10% of the positive samples. Only the HA gene region of influenza viruses was analyzed routinely for genotyping The one-RT-PCR products amplified with the primers described before were purified using Centri-Sep Columns (Princeton Separation; Englishtown, NJ) and sequenced using the BigDye Terminator v. 3.1 Cycle Sequencing Kit (Applied Biosystems; Foster City, CA) following the manufacturers’ instructions. Sequences were analyzed and edited using the Sequencer 4.8 software (Applied Biosystems; Foster City, CA).

Phylogenetic trees were constructed by the neighbor-joining method and bootstrap analysis to determine the best-fitting tree for the gene using MEGA software (version 4). The statistical significance of the tree topology was tested by bootstrapping (1,000 replicas). Pairwise distances between and within the genotypes at the nucleotide level were calculated with Kimura 2 parameters and with Poisson correction at the amino acid level with MEGA software. Genbank accession numbers are listed in Table S1.

### Statistical Analysis

Information on the CRFs was entered into a database created in Microsoft Office Access 2003. The chi square and Fisher exact tests were used to compare means and associations using SPSS software version 10.0 (SPSS Inc.; Chicago, IL) and R version 2.8.0 (R Development Core Team; Vienna, Austria).

## Supporting Information

Table S1 **Genbank accession numbers of DNA sequences from 27 Influenza viruses isolated in two health centers of Maracay, Venezuela: October 2006–December 2010.**
(XLS)
